# The development of a silage-based biorefinery to deliver the maximum nutritional benefit for human consumption from UK grasslands

**DOI:** 10.1098/rstb.2024.0161

**Published:** 2025-09-18

**Authors:** Sebnem Kurhan, Yubin Ding, Fatma Guler, Olusegun Abayomi Olalere, Samuel Eze, Anne Wambui Mumbi, Frank Vriesekoop, Helen Pittson, Bernardo Castro-Dominguez, Hannah Leese, Karl Behrendt, Richard Green, Christopher Chuck

**Affiliations:** ^1^ Department of Engineering, Harper Adams University, Newport, UK; ^2^ Department of Chemical Engineering, University of Bath, Bath, UK; ^3^ Department of Agriculture and Environment, Harper Adams University, Newport, UK; ^4^ Harper Adams Business School, Harper Adams University, Newport, UK; ^5^ Harper Food Innovation, Harper Adams University, Newport, UK

**Keywords:** grass, yeast, protein, mechanochemical, lipid, fermentation

## Abstract

In this work we aimed to increase the food potential of UK pasture by coupling targeted mechanochemical processing and novel biotechnology to convert silage into edible protein and lipid fractions. To this end, the water-soluble protein and vitamins were extracted from silage using a twin-screw extruder at room temperature. The extrusion of the silage was optimized in water with no additional chemicals. Under optimal conditions, 22 wt% of the silage was solubilized, with this fraction containing 52% of the protein present from the original material. The protein contained key essential amino acids with a profile similar to soy protein. Vitamins B_1_, B_2_, B_3_ (nicotinamide and nicotinic acid) and B_6_ (pyridoxine, pyridoxal and pyridoxamine) were also extracted. The resulting solids from the extruder, which contained further insoluble protein and the carbohydrates from the silage, were then depolymerized and used to culture the oleaginous yeast *Metschnikowia pulcherrima* producing further mycoprotein and lipid from the system. The mycoprotein contained a balanced amount of vital amino acids, while the yeast lipid had a fatty acid profile containing high levels of monounsaturated lipids. The silage was also found to contain high value lipids, rich in omega-6 linolenic acid. The work presented here represents a preliminary study but highlights the possibility of extracting edible nutrients from grass feasibly, with the potential to make UK agriculture far more resilient and sustainable.

This article is part of the theme issue ‘Transforming terrestrial food systems for human and planetary health’.

## 1. Introduction

The global food system is estimated to produce approximately 30% of all global greenhouse gas emissions [1,2]. Animal farming, especially raising cattle for meat and dairy, is responsible for over 60% of these related emissions [2,3] and produces the most carbon intensive sources of protein [4]. Within the UK, approximately 50% of the protein in the average diet comes from meat, with over 9 00 000 tonnes of animal proteins consumed. To support this industry, the UK’s grassland is used to grow approximately 9 000 000 cattle and 21 000 000 sheep [5]. As such grass is the UK’s largest agricultural crop with grassland covering approximately 12 M hectares (70% of agricultural land). It offers a substantial, significantly underutilized, potential future food source for the country. While a significant proportion of British grassland is in hilly terrain, estimates suggest over a third of the land (4 M+ hectares) is available for harvesting, which could conservatively produce over 20 M tonnes of grass, closely equivalent to the entire UK cereal output (approx. 22 M tonnes).

Despite the high nutritional content of grass, humans cannot digest the grass’s lignocellulosic structure and instead rely on animals to convert grass into digestible nutrient-rich foods such as meat and milk. This is an inefficient process, as cattle need up to 125 kg of fresh grass (20% dry weight) to produce 1 kg of edible meat or 5 kg of milk solids [6–9]. Grasses already contain relatively high amounts of protein, comprizing approximately 15% in fresh grass and between 11 and 15% in silage. If 20 M tonnes of grass was directly repurposed for human food, then this would equate to approximately 3 M tonnes of protein, approximately double the UK’s total protein consumption [10]. The major protein types in grasses are classified as storage and plasma membrane proteins, i.e. surface and globular proteins, and specifically ribulose biphosphate carboxylase/oxygenase (RuBisCO) which catalyses the solar energy into chemical energy through CO_2_ fixation [11] and also forms the edible protein corresponding to over 65% of the soluble leaf protein [12]. As such, grasses could make up a substantial protein source for human consumption with a far higher efficiency per hectare and producing just a fraction of the carbon emissions of traditional animal husbandry.

In addition to increasing domestic production of food, improving the utilization of grass will transform the UK food system through a host of other potential benefits. For example, making better use of existing grassland or introducing pasture leys into arable rotations enable land repurposing for environmental and other activities. As this technology is feedstock agnostic and will work with leguminous species and herbaceous meadow grass crops, it enables the reintroduction of flower-rich grasslands supporting a wide range of insects and fauna, increasing biodiversity aiding the resilience of current ecosystems. As grasslands are more tolerant to flooding, extreme weather and climate change, increasing grassland productivity will make the UK food system more resilient, sustainable and productive while reducing some of the negative impact of modern intensive agriculture on biodiversity.

To ensure an all-year-round supply of grass-derived feed for cattle, approximately 35% of the UK’s harvested grass is ensiled. A well-made and conserved silage serves as a year-round available source of digestible nutrients in ruminant diets, which reduces the risks of many diseases such as rumen acidosis and improves livestock growth and development [13]. Throughout the ensiling process, lactic acid bacteria start using free and bound carbohydrates in the biomass to produce lactic acid. The biochemical composition and quality of silage vary depending on the type of biomass used which may have implications for human nutrition [14]. The lactic acid preserves the grass by lowering the pH and as pH decreases, the rigid cell wall starts to partially hydrolyse which also enables more plant protein and digestible fibre to be extracted. Any technology aiming to produce food ingredients from grass would therefore need to use silage as the main feedstock which provides a relatively homogeneous feedstock supply through the various seasons of the year [13–15].

Selective, low-energy methods for protein extraction are vital to produce proteins from grass and silage on a large scale. However, conventional protein extraction methods are costly, energy intensive and use environmentally hazardous chemicals [16,17]. One promising alternative is mechanochemical-assisted extraction (MAE) that uses mechanical forces enabling low-energy, selective extraction of the target compounds [17,18]. Ball-mill and twin-screw extrusion are prominent systems with easy scale-up capabilities. Twin-screw extrusion is a continuous system combining thermal, chemical and mechanical practices, and hence reduces the amount of required reagents [19], unwanted oxidation and thermally induced reactions (i.e. Maillard reaction). Using MAE, it should be possible to break down the recalcitrant lignocellulosic fraction in the silage liberating the valuable, edible fractions of the cell.

While the proteins are a clear target for extraction, the major components in silage are cellulose and other bulk carbohydrates. A promising route to extract further value from these silage components is to use them as a feedstock to culture edible yeasts, capable of producing mycoprotein or myco-lipids from the resulting sugars. Yeast lipids have very similar fatty acid compositions to plant oils, with the lipid yield exceeding 50% of the cell dry weight depending on the yeast species [20]. The oleaginous yeast *Metschnikowia pulcherrima* is particularly promising for this application, owing to its ability to grow on a wide range of substrates under non-sterile conditions, at low pH and in the presence of fermentation inhibitors [21].

**Figure 1.**
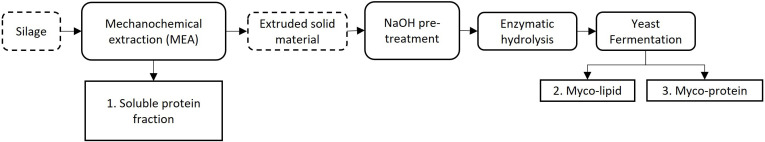
Flow chart demonstrating the ‘pasture to plate’ concept producing three food products from silage including a grass protein fraction with further soluble nutrients, a mycoprotein and myco-lipid fraction.

In this investigation, mechanochemical extraction was investigated as a low-energy method to extract the edible fractions from silage before culturing the yeast *M. pulcherrima* on the cellulosic fraction (figure 1). By converting the carbohydrate fraction with a yeast, this widens the scope of food products to include protein from yeast, and by using an oleaginous yeast, further lipids with alternative compositions.

## 2. Material and methods

### (a) Materials

Silage was obtained from the Crop and Environment Research Centre (CERC), Harper Adams University. The silage biomass from commercial Aber Red 5 (Germinal, UK) seed mix containing perennial ryegrass (*Lolium perenne*) varieties AberZeus, AberWolf, AberGain and red clover (AberClaret, *Trifolium pratense*) were fermented using Magniva Platinum (Lallemand, UK) silage additive. The oleaginous yeast, *M. pulcherrima* (NCYC4331), was used throughout.

The following reagents/materials from Sigma-Aldrich (St Louis, MO, USA) were used: the cellulase enzyme blend Cellic^®^ CTec2, acid phosphatase (AP), β-glucosidase (BGL) from almonds, pronase (PRO) from *Streptomyces grieus*, α-amylase (AMY) from *Bacillus* sp. sodium hydroxide, hydrochloric acid and anhydrous sodium carbonate nicotinic acid (B_3_), riboflavin-5-phosphate, reagent grade mono sodium phosphate. Thiamine hydrochloride (B_1_), riboflavin (B_2_), nicotinamide (B_3_), pyridoxine (B_6_) and pyridoxal hydrochloride (B_6_), pyridoxamine dihydrochloride (B_6_), 1-hexane sulfonic acid sodium salt and High Performance Liquid Chromatography(HPLC) grade methanol, phenol, sodium borate were obtained from Fisher Scientific (Loughborough, UK). Amino acid analysis was conducted on Agilent 1280 HPLC with a Diode-Array Detector (DAD) and a Fluorescence Detector (FLD) fitted with an Advanced Bio AAA, C18 analytical (4.6 × 100 mm, 2.7 µm) and a guard column (4.6 × 5 mm) (Agilent, USA). The derivatization reagents involving ortho-phthaldehyde/9-fluorenyl-methyl chloroformate (OPA/FMOC), amino acid standard mixes and borate buffer were supplied from Agilent (USA).

### (b) Methods

#### (i) Continuous extrusion

The extrusion of silage for extracting protein was performed with a Process 16 parallel twin-screw extruder (Thermo Fisher Scientific, Germany). An Aqua Pro chiller system was attached to modify the temperature of the barrel (Hitema, Padua, Italy). Further information on the extruder set-up is given in the electronic supplementary material. A single-factor protein yield analysis was first performed to determine the range for response surface methodology (RSM). The yield of protein was determined from the total nitrogen content. The extrusion parameters were selected based on a review of the existing literature [22,23]. In total, four factors were considered, including temperature (25–70℃), screw speed (40–100 r.p.m.), solid to liquid ratio (1 : 5–1 : 40) and salt addition (sodium carbonate, from 0.05 to 20%). Other factors were kept constant when analysing each factor. Afterwards, an RSM analysis was performed to optimize the protein yield with central composed design (CCD). The centre of the design space (alpha) was determined at 1.68. For non-centre points, extrusion was performed in duplicate while six independent extrusions were performed in the central point. Thus, in total, 34 tests were conducted with the pre-determined range (−1 to +1) of temperature (15–35℃), solid to liquid ratio (1 : 15−1 : 35) and screw speed (50–100 r.p.m.). Design Expert version 13.0.1.0 was used for the experiment design and figure drawing.

Mass balance calculations were performed to determine the yield of protein. A continuous 6 min extrusion was performed, and the solid residual was air dried in an oven at 55℃. Elemental analysis was performed to determine the protein content of the silage and silage residual. The yield and soluble rate were further calculated on a percentage basis.

#### (ii) Pre-treatment of silage and extruded material for culturing the yeast

Dilute alkaline solution (0.5% (w/v) NaOH) was used for pre-treatment of silage and the extruded material. The total solid loading ratio of the pre-treatment was 5% (w/v). All experiments were run for 100 ml in the 250 ml beaker on a hot plate at 75°C with vigorous mixing for 1 hour. Once samples were cooled to room temperature, and they were centrifuged at 3000 r.p.m. for 15 min. The solid fraction was washed with water until the pH turned neutral and then dried at 45°C overnight and stored at room temperature. The percentage of solid recovery was calculated on a dry basis over solid recovery as follows:


(2.1) solid recovery %=(WPRT / WUT)×100,

where WPRT is the weight of solid biomass recovered after pre-treatment (g) and WUT is the weight of untreated biomass subjected to pre-treatment (g).

Enzymatic hydrolysis was conducted in 50 mM sodium citrate buffer at pH 4.8 and 50°C in an incubator. The sample loading ratio was 8% (w/v), and the total reaction volume was 25 ml and conducted in a 50 ml falcon tube in 150 r.p.m. shaking conditions. The activity of Cellic CTec2 was determined according to the National Renewable Energy Lab (NREL) protocol [24] Cellulase activity was measured at 284.62 Filter Paper Units (FPU) ml^−1^, loading dose was 60 FPU g^−1^ biomass. Tetracycline with a 15 µg ml^−1^ concentration was added to the reaction to prevent microbial growth. The reaction was monitored for 96 hours and subsequently chilled to stop the enzymatic reaction. Samples were centrifuged at 6000 r.p.m. for 30 min and stored at −20°C. Enzyme and substrate controls were conducted for each run.

Silage hydrolysate, stored at −20°C, were defrosted at room temperature and filtered through a 0.2 µm Polyvinylidene fluoride (PVDF) filter to obtain a sterile sample. No other media components were added to the silage hydrolysate, and pH was not adjusted as it has been demonstrated previously that very similar biomass and lipid yields were achieved at pH 4 and 5 for *M. pulcherrima* [25]. The fermentations were performed in a 50 ml falcon tube with 2 ml hydrolysate as a working volume. *Metschnikowia pulcherrima* culture activated on soy malt broth was inoculated with a 2.5% (v/v) ratio of 24 hour active culture into the silage hydrolysate. Samples were incubated at 20°C at 200 r.p.m. for 140 hours. Initial screening growth of *M. pulcherrima* was monitored using optical density measurements at 600 nm in a spectrophotometer [26]. Measurement of dry cell weight (DCW) was done according to [27] at the end of the 140 hour incubation. *Metschnikowia pulcherrima* cells and lipid accumulation were observed under a light microscope (Olympus BX51) at 100× magnification [28]. Samples were also analysed for fermentable sugar depletion at the end of the 140 hour incubation.

#### (iii) Culturing *Metschnikowia pulcherrima*

*Metschnikowia pulcherrima* stock culture stored at −80°C was activated on malt extract agar (30 g l^−1^ malt extract, 5 g l^−1^ soy peptone, 15 g l^−1^ agar) at 25°C for 3 days [25]. A single colony was transferred into 20 ml soy malt broth (30 g l^−1^ soy peptone, 25 g l^−1^ malt extract, pH 5 with 6 M HCl) [25] and incubated at 25°C at 200 r.p.m. for 24 hours. *Metschnikowia pulcherrima* active culture was inoculated into silage hydrolysate. The yeast was cultured directly on the silage hydrolysate material produced from this process, without additional nutrients added, at 20°C over 140 hours. Throughout the fermentation, inoculum purity in silage hydrolysate was tested using the selective iron chloride added malt extract agar (15 g l^-1^ agar, 30 g l^-1^ malt extract, 5 g l^-1^ soy peptone, 0.02 mg l^-1^ FeCl_3_), on which *M. pulcherrima* forms pulcherrimin pigment enabling differentiation.

#### (iv) Analytical methods

Silage sample weights were calculated on a dry matter basis. The moisture content was determined by placing 1 g of the sample in an oven at 105°C and dried until the weight remained constant. Ash content was calculated after combusting the silage in pre-tared crucibles at 575°C for 6 hours with a 1°C min^-1^ ramp rate. Petroleum ether was used for the total lipid extraction in a FOSS ST 243 Soxtec F ^TM^ manual extraction system which was also used for determination of water and ethanol extractives in the silage.

The protein yield of the extruded sample was determined by total organic carbon (TOC) analyser (Shimadzu TOC-L-CPH/TNM-L). The extruded samples were filtered through a Millex-GV PVDF 0.22 μm membrane (Merck, Ireland) before being suitably diluted with deionised water. Potassium nitrate was used as a calibration standard which ranged between 10 and 1000 ppm. The total nitrogen and protein conversion factor used were 6.25 [29].

Total nitrogen content in the yeast broth was measured with CN828 249 model Leco carbon, hydrogen, nitrogen and protein determinator, and the 6.25 conversion factor was used to calculate the total protein content. Cellulose, hemicellulose, lignin and structural sugars content were analysed externally (Celignis Ltd, Ireland) according to NREL procedures [30].

The infrared spectra of the raw and alkaline extracts used in the pre-treatment of the biomass were obtained with a Bruker Fourier Transform Infrared Spectroscopy (FT-IR) INVENIO spectrometer in absorption mode over the range of 400−4000 cm^−1^. Thermogravimetric analysis of a 25 mg sample was applied as follows: a heating rate of 10°C min^-1^ from 50 to 900°C with a nitrogen flow at a rate of 50 ml min^-1^ using a Netzsch STA 449 F1 thermogravimetric analyser.

Lipid extraction of silage was completed according to Mir *et al.* [31] and lipids assessed through proton nuclear magnetic resonance spectroscopy (^1^H NMR) according to Chuck *et al.* [32]. Esterification of the lipids and screening for the fatty acid methyl esters profile using Gas Chromatography–Mass Spectrometry(GC-MS) was carried out according to ISO 12966−4 [33].

Silage amino acid composition and selected vitamin B vitamers quantities were determined. The amino acid extraction was performed according to the Dahl-Lessen *et al.* method [34] by hydrolysing the 20 mg of dry silage with 3 ml of 6N HCl containing 0.1% phenol in a glass headspace vial with crimp cap at 115°C for 24 hours. At the end of the digestion, vials were cooled down at room temperature, and caps were de-crimped and neutralized with 3 ml of freshly prepared 6N NaOH solution. After cooling, an aliquot of sample was filtered through a 0.22 µm nylon filter suitable for aggressive solutions (Fisher Scientific, UK) and diluted with 0.1N HCl. The tryptophan extraction method by La Cour *et al.* [35] was modified and performed. The silage sample (20 mg) in headspace vial was mixed with 3 ml of 95 mM ascorbic acid in 4.2N NaOH, tube content was flushed under an N2 stream and after crimping, the sample was digested at 115°C for 20 hours. After digestion, the sample was neutralized with 2 ml of 6N HCl and filtered into the HPLC vials. Hydrolysed samples were analysed with an Agilent 1280 Infinity HPLC-DAD/FLD system which enables us to set an injector method for FMOC-OPA in-column derivatization. The online derivatization and gradient analytical method were set according to the Agilent Advance Bio amino acid analysis kit instructions [36]. The samples were prepared in triplicate. Soy flour sourced from the National Institute of Standards and Technology (NIST3234) was used as the reference material to test extraction performance by comparing the results with the NIST3234 datasheet.

Vitamin B_1_, B_2_, B_3_ and B_6_ extraction from silage was completed through an adapted method given by Zhang *et al.* [37] and Salvati *et al.* [38]. Briefly, 30 mg of dry silage was dispersed in 270 µl of ultra-pure water and the pH adjusted to 6 with 15.5 µl of 1N NaOH, the tubes were then left to hydrate for 15 min at room temperature. After which the samples were placed onto a shaker set to 100°C at 1200 r.p.m. for 30 min, then cooled on ice and 80 µl of each AP (5 mg ml^-1^), AMY (20 mg ml^-1^), BGL (5 mg ml^-1^) and 30 µl of PRO enzyme solutions, which were prepared in water separately, were added, vortexed and incubated on the shaker set to 37°C at 1200 for 4 hours. After incubation, samples were centrifuged at 13 000*g* for 30 min and 200 µl of clear supernatant was transferred into a 10 kDa molecular weight cut-off spin filter (Vivaspin® 500, Sartorious, Germany) and centrifuged at 13 000*g*, at 4°C for 30 min to remove the enzymes. The filtrates were immediately transferred into HPLC vials with 200 µl inserts and injected to Agilent 1280 Infinity fitted with an Agilent Poroshell EC C18 analytical column (150 × 4.6 mm, 2.6 µm) using the gradient flow of mobile phase A (50 mM sodium hydrogen phosphate, 5 mM 1-hexane sulfonic acid sodium salt) and methanol (B) at 1 ml min^-1^ flow at 30°C.

Glucose, xylose, arabinose, cellobiose, formic, acetic and levulinic acid in the hydrolysate were analysed through an Agilent 1260 HPLC equipped with an refractive index (RI) detector and Aminex Bio-Rad HPX−87H column 300 × 7.8 mm. The mobile phase was 5 mM H_2_SO_4_ at an isocratic flow, at 0.6 ml min^-1^ with a 60°C column temperature and in 40℃ detector temperature. Defrosted samples were centrifuged at 13 500 r.p.m. for 5 min before analysis [39].

#### (v) Analysis of protein functionality

The foaming capacity (FC) of the silage protein fractions was determined using the protocol described previously [40,41]. Briefly, a 10 mg ml^-1^ protein suspension was prepared using 0.1 M phosphate buffer at pH 7.0. The volume of the suspension was measured both before and after homogenization and recorded as:


(2.2) $$FC=(\beta_{final}-\beta_{initial})/\beta_{initial},$$

where *β*_final_ represents the post-homogenization volume in millilitres, and *β*_initial_ denotes the starting volume of the buffer suspension in millilitres.

Furthermore, the method used by Tan *et al.* [41] was replicated to determine the emulsifying capacity of the protein fraction. The emulsifying activity was estimated as a ratio of emulsion layer and post-centrifugation volume, such that:


(2.3) $$emulsifying\;activity\;(\%)={Vol}_{\left(emulsion\;layer\right)}/{Vol}_{\left(Final\;volume\right)}\times 100,$$

where Vol_(Final volume)_ is the post-homogenization total volume while Vol_(emulsion layer)_ is the volume of emulsion layer only.

Additionally, the stability of the emulsion layer was assessed by incubating it in a hot water bath at 80°C for 30 min to denature the protein. The emulsifying stability was then determined using the equation below, immediately after centrifuging for 5 min


(2.4) $$emulsifying\;stability\;(\%)=100-[S_{\left(after\right)}/S_{\left(initial\right)}\times 100],$$

where *S*_after_ represents the volume of the emulsion after a 30 min incubation at 80°C, and *S*_initial_ denotes the starting volume of the emulsion layer prior to heating.

## 3. Results and discussion

### (a) Edible components of the silage feedstock

This study selected a mix of perennial ryegrass-red clover silage as a representative grass feedstock for the UK. The silage used has a relatively high crude protein content (15.2%), which is consistent with the values reported in other studies [40]. Previous studies have mostly focused on the performance of livestock fed with ryegrass-red clover silage in terms of nutrient utilization and product yield such as milk, with variable results reported [42,43]. This line of research is important as livestock is known to convert biomass that is not suitable for direct human consumption into important food [44]. However, the prospect of extracting food components directly from biomass without prior livestock conversion is an exciting prospect. Despite the nutrient losses often associated with silage-making [45], the ryegrass-red clover silage used in this study still has valuable nutrients such as carbohydrates, protein, lipids and vitamins that can be extracted via mechanochemical methods (table 1).

**Table 1.** Composition of silage feedstock used in this study; all values were experimentally determined.

ash (g 100 g^−1^)	lignin and carbohydrates (g 100 g^−1^)		lipids (g 100 g^−1^)		proteins (g 100 g^−1^)		vitamin B (mg 100 g^−1^)	
11.73 ± 0.09	lignin	12.15 ± 0.25	total lipid (sum of fatty acids)	1.71 ± 0.01	crude protein	15.16 ± 0.2	thiamine	0.00 ± 0.00
	cellulose+hemicellulose	32.65 ± 0.01	**Fatty acid profile (% of glyceride lipid**)		**amino acid profile**		riboflavin	0.093 ± 0.04
			palmitic acid (16:0)	19%	aspartic acid	1.11 ± 0.0	nicotinamide	19.92 ± 3.94
	glucan	20.62 ± 0.02	stearic acid (18:0)	3%	glutamic acid	1.30 ± 0.0	pyridoxal	11.88 ± 0.21
	xylan	9.57 ± 0.01	oleic acid (18:1) (n−3)	3%	serine	0.41 ± 0.0	pyridoxamine	263.34 ± 8.99
	mannan	0.17 ± 0.01	linoleic acid (18:2) (n−6)	12%	histidine	0.19 ± 0.0	pyridoxine	1.86 ± 0.01
	arabinan	1.58 ± 0.00	linolenic acid (18:3) (n−3)	49%	glycine	0.77 ± 0.0		
	galactan	0.59 ± 0.00	eicosenoic acid (20:1) (n−9)	2%	threonine	0.47 ± 0.0		
	rhamnan	0.12 ± 0.00	lignoceric acid (24:0)	2%	arginine	0.39 ± 0.0		
			nervonic acid (24:1) (n−9)	2%	alanine	1.12 ± 0.0		
			others	9%	tyrosine	0.32 ± 0.0		
					valine	0.81 ± 0.0		
					methionine	0.23 ± 0.0		
					tryptophan	0.02 ± 0.0		
					phenylalanine	0.53 ± 0.0		
					isoleucine	0.52 ± 0.0		
					leucine	0.91 ± 0.0		
					lysine	0.59 ± 0.0		

For example, the silage was found to contain important vitamins especially vitamins B_3_ (nicotinamide) and B_6_ (pyridoxamine), used in dietary supplements for addressing age-related illnesses [46]. The inedible lignin only accounts for approximately 12% of the grass feedstock. Carbohydrates such as cellulose make up over half of the mass. Interestingly glucans, which are increasingly being used as an essential ingredient in diets to improve palatability and reduce the risks of heart diseases and diabetes, are present in high levels (20.6%) [47,48]. This suggests that silage has the potential to serve as a ready source of an important dietary fibre fraction with great health benefits, as well as a feedstock for further biochemical conversion.

Unlike the carbohydrates, lipids account for only 1.7% of the silage. Despite this small proportion, the silage lipids consist mainly of polyunsaturated fatty acids or ‘healthy fats’, which are essential supplements in human diets. For example, linolenic acid (18:3, n-3) makes up 49% of the silage lipids and has been shown to be highly oxidizable, making it an essential ingredient for addressing health problems associated with lipid consumption such as obesity [49]. Linolenic acid is relatively rare in terrestrial oils, with linseed oil being one of the few sources of this ester. Grass could therefore be a viable source of this highly valuable fatty acid.

**Figure 2.**
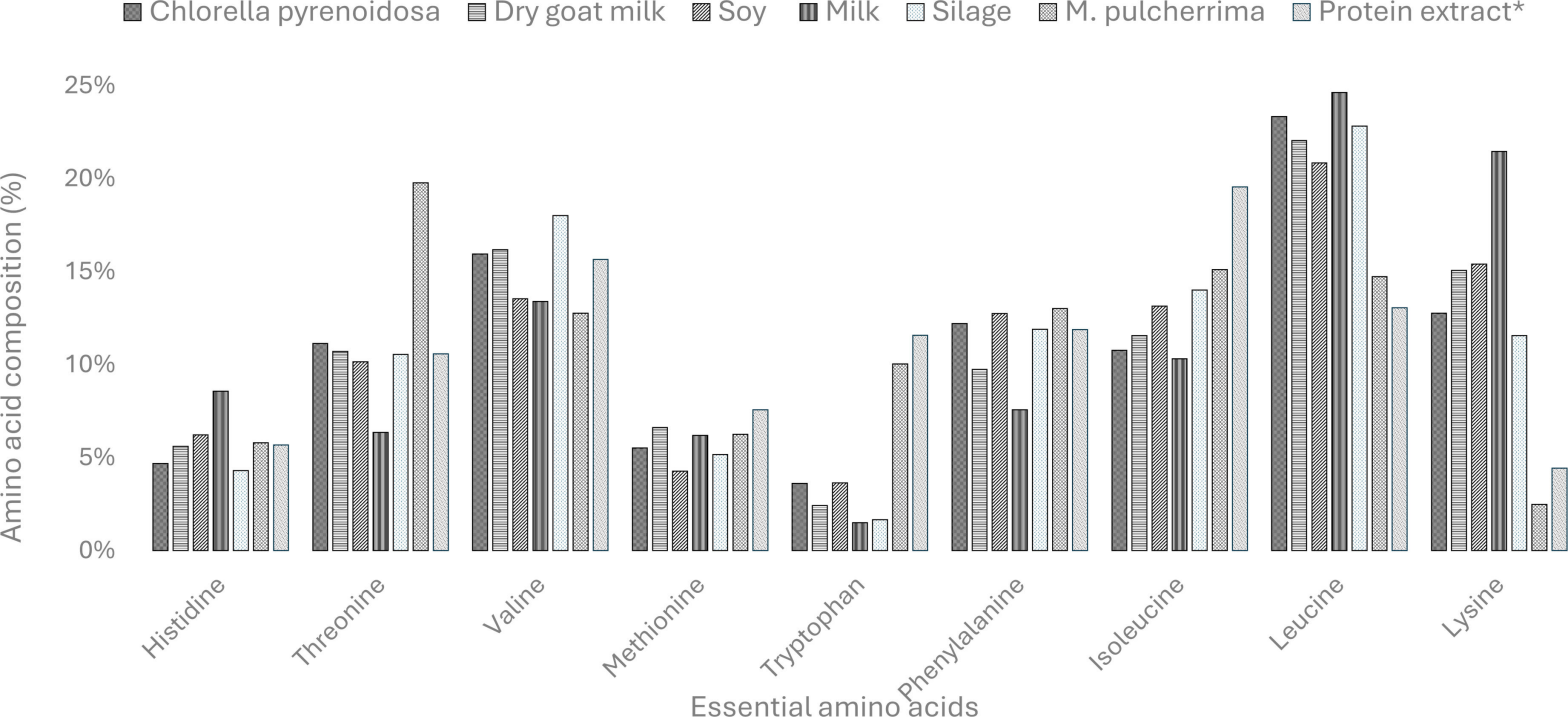
The amino acid composition of the essential amino acids, as a percentage of the total essential amino acids, for the silage used in this study, protein extract of silage (*), *M. pulcherrima* and examples of staple and alternative proteins.

The main application of silage for human consumption is as a protein source. A detailed analysis reveals both its strengths and areas where it might be complemented by other proteins. For instance, the amino acid profile of silage (figure 2) contains seven essential amino acids, positioning it as a comprehensive and nutritionally valuable protein source, comparable to soy protein. Silage appears as a promising alternative to conventional plant-based proteins, particularly in the context where a broad spectrum of essential amino acids is desired. The analysis also shows that silage has relatively low levels of histidine compared to other proteins, which can be compensated by combining it with the mycoprotein from *M. pulcherrima*, known for its higher histidine content [50]. Additionally, while silage is well-represented in isoleucine, leucine, threonine and valine; amino acids critical for protein synthesis and muscle repair [51], it has slightly reduced levels of lysine compared to milk and soy protein. Lysine is vital for various metabolic functions, and although silage provides a sufficient amount, supplementation with other lysine-rich proteins might be necessary in certain applications. Moreover, the phenylalanine content in silage is lower than in wheat, which could be a consideration depending on specific nutritional needs [52].

In terms of functionality, protein extracted from silage exhibited a relatively low FC, below 20%. This is not comparable to other protein functional sources such as egg white protein, whey protein and sesame protein which can be as high as 200%. This low capacity indicates that silage is not ideal for applications that require significant foaming ability, such as in the production of meringues, mousses and whipped toppings [53,54]. Instead, the protein content of silage is more suited for use as a bulk protein substitute in food products where foaming properties are not a priority, making it a practical choice for formulations that focus on protein enrichment rather than aeration. Similarly, the emulsion capacity and stability of protein from silage are also relatively low, below 20%. Its emulsion capacity is similar to that of soy protein, but both capacity and stability are significantly lower than those of proteins like wheat germ, which is typically over 100% demonstrating superior performance in maintaining stable emulsions. This suggests that while protein extracted from silage can form emulsions, it may not be as effective in applications where high emulsion stability is crucial, such as in salad dressings, mayonnaise and sauces [55].

### (b) Mechanochemical extraction of soluble proteins from silage

**Figure 3.**
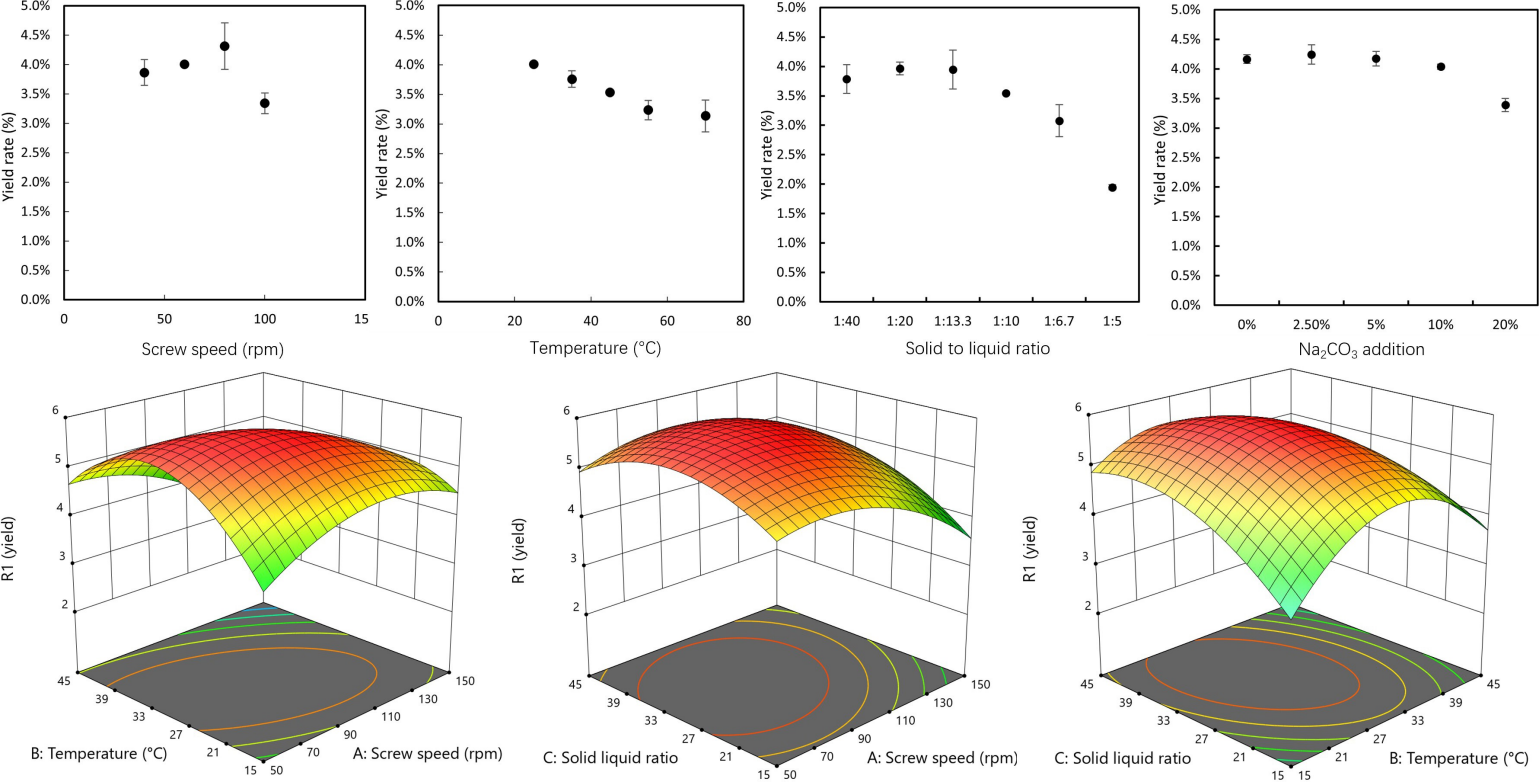
The single-factor analysis (A) and response surface plot (B) of the silage extrusion for protein extraction.

To extract the protein from silage effectively, the silage was processed using a twin-screw extruder. Figure 3 shows the single-factor analysis conducted over the four parameters, indicating that three of them have a significant influence on the final protein yield. The most influential factor was the solid to liquid ratio, displaying the biggest protein yield gap of 2.2% dried weight. Meanwhile, the influence of temperature (yield gap of 0.9%) and screw speed (yield gap of 1.0%) were also found to be significant. The addition of Na_2_CO_3_ was found to have negligible effect on the final yield of protein (4.2% yield achieved with 0, 2.5 and 5% concentration of Na_2_CO_3_), despite being demonstrated previously to enhance extraction of protein from grasses [17].

The extraction results were similar to previous reports on extrudermediated extraction of protein from plants. For example, extraction from alfalfa resulted in a lower solid to liquid ratio and increased final protein yield [22]. Where the authors reasoned that the water introduced to the barrel is able to increase the washing action and aids solubilizing the protein from the silage, higher levels of water also decreased shear and pressurizing forces required to disrupt the structure of biomass during extrusion, thus decreasing the solubility of the material. Here, water acts as a lubricant that prevents structural breakage, thus a careful balance must be struck to maximize solubilization while reducing lubrication.

Temperature also affects the extraction of protein from grass. At low temperatures, lignocellulosic materials tend to become more brittle. The reduction in temperature potentially reduces the mobility of the polymer chains within the lignin, cellulose and hemicellulose components of silage. This decreased molecular motion would then make the material more rigid and prone to cracking or breaking under stress, thus facilitating extraction. Essentially, the material’s toughness decreases, and it becomes easier to fracture and extract the protein. On the other hand, at high temperatures, silage typically becomes less brittle and more ductile, hindering the extraction of protein.

The effect of screw speed also influences protein extraction. At higher screw speeds, the material experiences more intense shear and impact forces as it moves through the extruder. These forces are crucial for breaking down the fibrous structure of silage. The increased mechanical energy input enhances the disruption of the cellulose, hemicellulose, and the lignin matrix, leading to greater breakage and size reduction of the material. Moreover, higher screw speeds also improve the mixing within the barrel, thus resulting in a more homogeneous mixture and potentially better access to the proteins. It is important to note that excessively high screw speeds can lead to overprocessing, where the material is subjected to too much mechanical stress. This can cause undesirable effects, such as excessive heat generation, which may lead to thermal degradation [23].

Since the protein extraction factors can significantly influence the final yield, an RSM was performed to determine the optimal conditions for the extrusion. The influencing factors and their range, screw speed (A, 50−100 r.p.m.), temperature (B, 15−35 ℃) and solid to liquid ratio (C, 1 : 15−1 : 35) were determined from the single-factor analysis. A CCD model was used, and the ANOVA of the model is provided in the electronic supplementary material. A quadratic model was suggested, and the *F*-value of 31.15 (*p*‐value < 0.001) implies the model is significant. The ANOVA analysis of the quadratic model shows that the correlation between temperature and screw speed (AB, *p*‐value = 0.0086), temperature and solid to liquid ratio (BC, *p*‐value = 0.0181) were high. Meanwhile, the square of all three factors are significant model terms (*p*‐value A^2^ = 0.04, B^2^ < 0.01 and C^2^ < 0.01). The three-dimensional response surface plots for protein yield are given in figure 3, with the interactions of each two variables shown when the third variable is set at the middle level presented. Accordingly, the optimized condition for the highest protein yield was found to be 28℃ with a screw speed of 82 r.p.m. and solid to liquid ratio 1 : 31. With the optimized condition, the protein yield was calculated to be over 50% of the total in the biomass.

The calculated optimized conditions were applied and the mass balance for the system completed (table 2). The protein content of the residual silage was calculated and compared with the protein content in the solubilized extract. A total of 52% of protein was extracted and distributed into the water phase. To achieve this only 22% of the total silage was solubilized, resulting in 78% of the material retained as a solid for further processing. This is a similar extraction rate and protein content to that extracted from alfalfa using extrusion [22,56] and for soybean and sunflower meal [57,58].

**Table 2.** The mass balance calculation of protein extracted from silage with extruder.

	% of total weight
silage protein in the extract (as function of total protein)	52%
residual protein in solid material	48%
total silage material solubilized (as function of dry weight added)	22%
protein content in the soluble fraction (calculated from model)	26%
protein content in the soluble fraction (actual)	25%

While the application of extruders for protein extraction is less common in the food industry than the use of ball milling, maceration or enzyme hydrolysis [59], extrusion is eminently scalable and provides a continuous method for handling large volumes of materials with minimal energy input [60]. In addition, similar levels of protein can be extracted from the material while removing far less of the additional unwanted products when compared to laboratory scale ball mills. For example, in this study, 22% total solubles were extracted with the extruder, removing 52% of the protein present. This compares favourably to 45% total solubles extracted by the ball milling of ryegrass which removed 63% of the total protein [17]. This more efficient extraction is presumably owing to more polysaccharides or minerals being dissolved in the liquid with the longer grinding time (usually 10−30 min), while the short retention time for extrusion (1–5 min) avoids any further solubilization.

### (c) Oleaginous yeast culture on the silage hydrolysate

On processing the silage, the solid material retained from the extruder was then further assessed as a feedstock for the yeast *M. pulcherrima*. However, the yeast was found to be unable to break down either silage, or the processed material recovered from the extruder without further treatment (table 3).

**Table 3.** Sugar and yeast yields from the various silage processes.

		silage with no extrusion processing			solid material from extruded silage		
		no processing	enzymatic treatment only	pre-treatment and enzymatic	no processing	enzymatic treatment only	pre-treatment and enzymatic
mass loss on NaOH pre-treatment %		—	—	47.4%	—	—	24.9%
total sugars released (g l^−1^)		<1	17.90	46.15	<1	15.5	42.5
	glucose	<0.1	13.74	32.75	<0.1	10.66	26.49
	xylose	<0.1	4.16	11.93	<0.1	4.50	14.35
	arabinose	<0.1	<0.1	1.47	<0.1	0.33	1.65
TOC (mg l^−1^)			28 005	29 567		11 321	18 880
TN (mg l^−1^)			1,590.75	534.63		713.13	638.43
TOC/TN^a^		—	17.60	55.28	—	15.9	29.6
yeast biomass (g l^−1^)		—	10.6	20.5	—	6.4	11.6
Y_m/m_^b^		—	0.59	0.44	—	0.41	0.27

^a^ The ratio of total organic carbon (TOC) to total nitrogen (TN)

^b^ Y_(m/m)_ is the mass percentage of yeast biomass achieved per unit of metabolizable sugar.

#### (i) Effect of pre-treatment on the silage

To increase the biomass accessibility and digestibility for hydrolytic enzymes, the selective degradation of the aryl ether linkages in lignin is necessary to increase the hydrophilicity of the lignin. The use of sodium hydroxide as a pre-treatment, together with solvation of the hydroxyl groups also swells the carbohydrate [61].

To this end, alkaline pre-treatment was employed to break the bonds between xylan and lignin, as well as the acetyl groups of the hemicellulose [62]. In this study, 0.5% sodium hydroxide solution was applied to both silage and the extruded solid material to deconstruct the lignocellulosic structure. The pre-treatment stage was assessed through FT-IR, and changes to the functional group regions (2852, 2918 cm^-1^) and the fingerprint (1800−800 cm^-1^) suggested large structural changes in the silage (see the electronic supplementary material). On pre-treatment 47.4% of the material was lost in the fresh silage sample, and 24.9% in the extruded material. While this represents a significant mass loss, this is in line with other studies in using NaOH as a preliminary pre-treatment [62,63]. The reduced mass loss for the extruded sample reflects that the more labile components have already been removed in the extrusion stage.

#### (ii) Enzymatic hydrolysis

To release the sugars from the carbohydrate, an enzymatic hydrolysis step was investigated using the CTEC2 enzyme mix. The silage and the extruded material both demonstrated some limited sugar release without pre-treatment, with 17.9 g l^-1^ and 15.5 g l^-1^ total sugars produced, respectively. However, on pre-treatment of the biomass this was increased to 46.2 g l^-1^ and 42.5 g l^-1^, respectively. The major monosaccharides released were glucose, xylose and arabinose. Plant hemicelluloses contain two main monosaccharides; xylose and arabinose with glucose being predominantly produced from cellulose [63]. The presence of these sugars in the extruded material suggests the incomplete degradation of the hemicellulose in the initial silage extrusion. The TOC content was found to vary between samples, with over 29.6 g l^-1^ TOC for the silage hydrolysate, but only 18.8 g l^-1^ for the extruded material. While the latter number comes almost entirely from the monosaccharide content of the solution, there is significantly more carbon in the hydrolysate produced from the silage, without an extrusion step, as there is in the solubilized monosaccharides in the sample. This is presumably owing to the further processing of the extruded samples and therefore extraction of more soluble organics in the extrusion stage.

Alkaline pre-treatment results in the release of nitrogen from plant cell walls which decreases the nitrogen content of biomass [64,65]. For this reason, the carbon/nitrogen (C/N) ratio of biomass hydrolysate depends on the loss that occurred during the pre-treatment as well as the initial nitrogen amount, the enzyme dose and the specific activity [66]. The total nitrogen content of the treated silage hydrolysate and extruded material hydrolysate were found to be 534.6 mg l^-1^ and 638.4 mg l^-1^ after 96 hour enzymatic hydrolysis, respectively. This fell within the suitable range for *M. pulcherrima* [25], and the hydrolysates produced were then used as media in the yeast fermentation directly.

#### (iii) Yeast culture on the silage hydrolysates

The yeast was cultured directly on the material produced from this process, without additional nutrients added, at 20°C over 140 hours (table 3). *Metschnikowia pulcherrima* can metabolize most C_5_ and C_6_ monosaccharides and shows good fermentation performance on DP2 and higher polysaccharides [67,68]. Consumption of all the monosaccharides was also observed for *M. pulcherrima* growth in the hydrolysates during 140 hour fermentation. The highest DCW was achieved on the silage material pre-treated with NaOH and processed with enzymes which also consisted of the highest amount of fermentable sugar. The fermentation is very efficient with a Y_M/M_ similar to strains grown on optimized synthetic medias. The yeast also produced 40% w/w in lipid, similar to that on synthetic media [25].

Although the hydrolysate produced from the processed extruded silage showed similar fermentable sugar yields and an appropriate amount of nitrogen, the yeast grew less efficiently on the extruded material with only 11.6 g l^-1^ DCW achieved, a Y_m/m_ of 0.27. These titre and efficiency were more in line with previous reports culturing the yeast on complex lignocellulosic hydrolysates [66]. This suggests that while the yeast can grow on C5 sugars, this is less efficient, and the elevated glucose and additional carbon in the silage hydrolysate seems to support better growth. The yeast grown on the material that was not pre-treated but solely degraded by enzymes grew efficiently but produced low levels of lipid. Presumably, this is owing to the low C/N ratio, as previously it has been reported that lipid accumulation is induced only when the C/N ratio is higher than 20 [69].

The fatty acid profile was assessed for the final lipid produced from yeast cultured on the extruded material (see the electronic supplementary material) and found to be predominantly monounsaturated, in line with previous reports for this organism [25]. This work demonstrates that while the silage carbohydrates are suitable for fermentation and can be converted efficiently to yeast biomass, multiple processing stages, which require further optimization are necessary to release the nutrients needed to culture the organisms.

### (d) Overall assessment of the process, further work and challenges

In this publication, a novel bioprocess for extracting edible fractions from silage is presented. In this system, over 50% of the protein in the silage was extracted in a solvent-free extrusion process, before the carbohydrates were further processed into a suitable fermentation media to culture the oleaginous yeast *M. pulcherrima*. While the silage protein was relatively easy to remove, the remaining protein and lignocellulosic structure was much harder to access and had to be pre-treated with alkaline, prior to enzymatic treatment, to produce a suitable media for yeast cultivation. On processing, the resulting hydrolysate was found to be suitable for cultivation with no further additional nutrients needed to achieve yeast growth. While the bioprocess would need further development, as large mass losses were observed in these stages reducing the yield of the system substantially, the nutrients released from the system were found to be suitable feedstocks to support myco-lipid production. In further iterations of the system, either the carbon lost needs to be recovered in another process, such as anaerobic digestion, or more efficient pre-treatment stages investigated.

In addition to protein and yeast lipid, various other high value components were identified from the system that could potentially be accessed as part of this process. While silage was found to have very low amounts of lipid, the level of omega−6 linolenic acid accounts for over 50% of the total, giving this a far higher value than standard commodity plant oils. Therefore, in the next iteration of this system, there is the potential to combine the lipid extraction of both yeast and grass to produce a high value nutraceutical oil product. Similarly, another source of value is in the range of B vitamins present in silage that are extracted alongside the soluble protein. For example, silage is rich in vitamin B_6_, which is beneficial for overall human health. Therefore, further experiments focusing on the vitamin B content in the extracted protein fractions will be beneficial to produce further value from the system.

In order to be able to scale the system, an appropriate extraction method is key. In comparison to other extraction methods (i.e. organic solvents or supercritical CO_2_ for example), the extrusion process is eminently scalable and effects the extraction by employing mechanochemical forces in a continuous process, ideal for high throughput industrial processing. Further work will focus on the design of the screw profile to increase the yield of protein while keeping the total solid yield constant.

To improve current food systems via novel biorefineries, it is vital to assess the whole system suitability for large-scale production including the capital and operating costs as well as the environmental impact of the products. To this end, future work will evaluate the required inputs and achieved outputs via technoeconomic modelling and the overall sustainability assessed through life cycle analysis to understand the process dynamics, pinch points as well as the overall cost and environmental impacts. This will lead to further enhanced design and the necessary unit operations to form the most productive system.

A further challenge comes in the application of these materials in the food sector. While the methods presented have been successful in extracting the bulk nutritional material, the next challenge is how to purify extracts to remove undesirable sensory and health attributes. Silage has volatile and non-volatile compounds such as lactates, terpenes and esters, which are sometimes produced or modified during the ensiling process [15] and affect the colour, taste and smell of nutrient extracts. Further research will therefore be focused on understanding how best to increase the yield and quality of nutrients extracted from silage to ensure that they have desirable sensory and functional properties for human consumption. In addition to the optimization of silage making, the method of nutrient extraction needs to be carefully selected and optimized as this has been shown to affect the functional properties of nutrient extracts [70].

The success of the work inherently lies in the acceptance and use of these food products in human diets. Our previous study revealed that the acceptance of grass-derived ingredients among UK consumers may face several challenges [71]. A major barrier is the lack of knowledge about this relatively new concept and the technology behind it, without sufficient understanding, consumer confidence in grass-derived products remains low. Therefore, additional educational strategies should be investigated to raise awareness, potentially through recipes and practical examples showcasing the use of these products, while emphasizing their health benefits as recommended by [72]. Changing attitudes and social norms surrounding these ingredients also poses a challenge, as many consumers may be hesitant to embrace unfamiliar foods. Ensuring the public is adequately informed about the sustainability and environmental benefits of grass-derived products are another obstacle that may correct these attitudes. Additional findings reveal that product design must carefully address concerns about health, safety and nutrition to gain consumer trust as these are key issues that matter most to consumers.

In this study, promising preliminary results demonstrated that protein and water-soluble vitamins could be extracted in a low-energy, scalable continuous process, with the remaining silage biomass being demonstrated to be suitable for further bioprocessing. If successfully brought to market, then this approach has the potential to increase the food potential of UK pasture, converting grass directly into nutritious edible fractions for healthier and more affordable alternative foods, making UK agriculture more resilient and sustainable.

## Data Availability

All primary data presented in this study are available from the University of Bath online repository https://doi.org/10.15125/BATH-01534. Supplementary material is available online [73].
